# The Core-Clock Gene *NR1D1* Impacts Cell Motility In Vitro and Invasiveness in a Zebrafish Xenograft Colon Cancer Model

**DOI:** 10.3390/cancers12040853

**Published:** 2020-04-01

**Authors:** Alireza Basti, Rita Fior, Müge Yalҫin, Vanda Póvoa, Rosario Astaburuaga, Yin Li, Julian Naderi, Miguel Godinho Ferreira, Angela Relógio

**Affiliations:** 1 Institute for Theoretical Biology (ITB), Charité—Universitätsmedizin Berlin, corporate member of Freie Universität Berlin, Humboldt—Universität zu Berlin, and Berlin Institute of Health, 10117 Berlin, Germany; 2 Molecular Cancer Research Center (MKFZ), Medical Department of Hematology, Oncology, and Tumor Immunology, Charité—Universitätsmedizin Berlin, corporate member of Freie Universität Berlin, Humboldt—Universität zu Berlin, and Berlin Institute of Health, 10117 Berlin, Germany; 3 Champalimaud Centre for the Unknown, Department of Experimental Clinical Research, Lisbon 1400-038, Portugal; 4 Institute for Research on Cancer and Aging of Nice, INSERM U 1081, CNRS UMR7284 UNS, Université Côte d’Azur, 06107 Nice, France; 5 Institute of Systems Medicine and Bioinformatics, Department of Human Medicine, MSH Medical School Hamburg—University of Applied Sciences and Medical University, 20457 Hamburg, Germany

**Keywords:** circadian clock, colon cancer, zebrafish xenograft, micrometastasis, apoptosis, proliferation

## Abstract

Malfunctions of circadian clock trigger abnormal cellular processes and influence tumorigenesis. Using an *in vitro* and *in vivo* xenograft model, we show that circadian clock disruption via the downregulation of the core-clock genes *BMAL1*, *PER2*, and *NR1D1* impacts the circadian phenotype of *MYC*, *WEE1*, and *TP53*, and affects proliferation, apoptosis, and cell migration. In particular, both our *in vitro* and *in vivo* results suggest an impairment of cell motility and a reduction in micrometastasis formation upon knockdown of *NR1D1*, accompanied by altered expression levels of *SNAI1* and *CD44*. Interestingly we show that differential proliferation and reduced tumour growth *in vivo* may be due to the additional influence of the host-clock and/or to the 3D tumour architecture. Our results raise new questions concerning host–tumour interaction and show that core-clock genes are involved in key cancer properties, including the regulation of cell migration and invasion by *NR1D1* in zebrafish xenografts.

## 1. Introduction

Cellular homeostasis is significantly altered in cancer, enabling abnormal proliferation and metabolic alterations to take place [1]. The complementary escape from immune surveillance, as well as resistance to apoptosis allows for further malignant cellular proliferation and metastasis to occur [1]. These different aspects constitute the hallmarks of cancer [1] and mounting evidence points to their regulation via the circadian clock [2]. The circadian clock generates ~24-hour rhythms in gene and protein expression, physiology, and behaviour [3,4,5] and in mammals, malfunctions of the circadian system have been reported to be associated with cancer and may be relevant for therapy [6,7,8,9,10]. Molecular clocks are formed by the heterodimer complex CLOCK/BMAL1 that binds to E-box elements in the promotor region of the genes Period (*PER1, PER2, PER3*) and Cryptochrome (*CRY1, CRY2*), as well as the nuclear receptors genes *NR1D1, NR1D2* and *RORα, RORβ, RORγ* activating their transcription via interconnected positive and negative transcriptional/translational feedback loops. This core-clock network (CCN) [11] generates ~24 h rhythmic oscillations in its target genes, the clock-controlled genes (CCGs), several of which are involved in the cell cycle, proliferation and metabolism and alterations in their expression are associated with cancer onset and progression (reviewed in [9]). Examples of such CCGs are *MDM2* and *VEGF*, involved in proliferation and angiogenesis [12,13], the glycolytic enzyme pyruvate kinase [14] and *HKDC1* involved in metabolism [15], and the cell cycle check point regulators *WEE1*, *MYC*, *CDKN2* and *cyclin D1* [11,16,17]. Several studies have shown a bidirectional interplay between cell cycle and the circadian clock in proliferating cells (reviewed in [18]). In the absence of external resetting cues, the circadian clock is phase-locked with the cell cycle in a 1:1 ratio, with *NR1D1* reporter expression reproducibly peaking 5h after mitosis [19]. Interestingly, pharmacological activation of *NR1D1* and *NR1D2* is specifically lethal to cancer and senescent cells, with no effect on viability of normal cells or tissues [20]. Moreover, PER1 interacts with proteins involved in DNA damage response and *PER1* overexpression has been shown to suppress the growth of human cancer cell lines [21]. p53, a key element in mediating DNA damage response and cell cycle binds to a response element in the *PER2* promoter region, which overlaps with an E-box and inhibits CLOCK/BMAL1-mediated transcription [22]. Another CCG, the histone deacetylase sirtuin 1 (SIRT1) can both promote or suppress tumour growth, depending on its interacting partners [23]. Altogether, these results suggest the existence of a direct link between transformation and perturbations of core-clock genes. 

Here we set up to investigate the putative impact of a dysregulated clock in a colorectal cancer (CRC) model *in vitro* and *in vivo* in zebrafish larvae xenografts, which have become an emerging model for *in vivo* and single cell cancer studies [24]. Among the several comparable model organisms suitable for *in vivo* approaches (e.g. chick embryo and mouse), zebrafish offers accessible single cell analysis and the possibility to quantify the impact of molecular alterations of circadian clock components in tumorigenesis (tumour size and apoptosis). The zebrafish embryo only develops an effective adaptive immune system after 9–12 days post-fertilisation, which makes it a suitable model for (xeno)-transplantation experiments. Furthermore, the zebrafish embryo model (in comparison to e.g. chick embryo model) offers the power of replicates, which is not available within other comparable model organisms. Thus, our *in vivo* data is only surpassed by our *in vitro* system in that regard. We generated CRC knockdown (KD) cell lines for different core-clock genes and analysed their impact in terms of proliferation, apoptosis, and migration. As an *in vitro* model system, we used HCT116 and SW480 colon cancer cell lines, which have robust circadian rhythms, as previously reported, and we are familiar with their cellular properties and genetic background [15,25]. Additionally, successful chronotherapy data has been reported for patients with colon cancer, and published clinical studies that attempted to fit the therapy to the individual’s clock properties show a positive impact in decreasing side effects and/or increasing survival (reviewed in [26,27]). Our data points to a role of circadian disruption in proliferation, apoptosis, and migration in CRC cells both *in vitro* and *in vivo* and highlights a function for the nuclear receptor, and core-clock element, NR1D1 as an enhancer of cancer invasiveness.

## 2. Results

### 2.1. The Knockdown of Core-Clock Genes Affects the Oscillatory Phenotype of HCT116 Cells In Vitro

**Figure 1:**
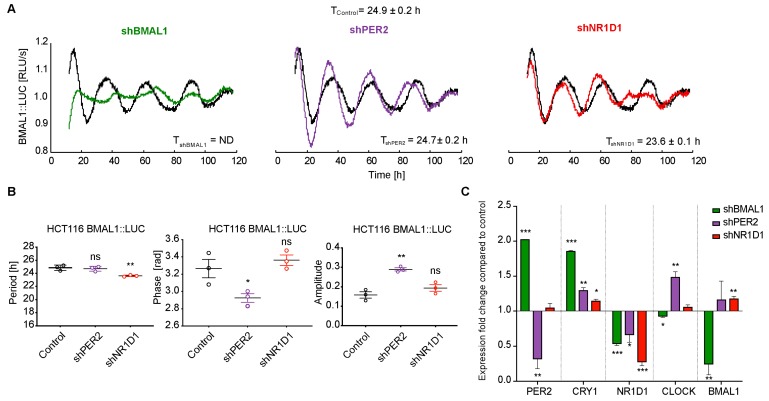
*BMAL1* promoter activity shows different oscillation patterns in HCT116 knockdown cell lines. (**A**) Bioluminescence readouts for the promoter activity of *BMAL1* over the course of 120 h in HCT116 control and knockdown (*shBMAL1*, *shPER2* and *shNR1D1*) cell lines. Periods were calculated with ChronoStar software (T_Control_ = 24.9 ± 0.2 h, T*_shBMAL1_* = ND, T*_shPER2_* = 24.7 ± 0.2 h, T*_shNR1D1_* = 23.6 ± 0.1 h, *n* = 3, mean ± SEM). (**B**) Period, phase and amplitude analysis of circadian bioluminescence data of HCT116 knockdown cells over the course of 120 h using Chronostar. (**C**) Gene expression analysis of core-clock genes *PER2*, *CRY1*, *NR1D1*, *CLOCK*, and *BMAL1* in HCT116 control and knockdown cell lines at 24h after synchronisation. ND, not defined, ns or no asterisk *p* > 0.05, * *p* < 0.05, ** *p* < 0.01, *** *p* < 0.001; two-tailed unpaired *t*-test.

In order to assess the impact of perturbing core-clock elements in the clock phenotype, we analysed the rhythms of *BMAL1* and *PER2* promoter activity in HCT116 control and knockdown (KD) cell lines (sh*BMAL1*, sh*PER2* and sh*NR1D1*) in real-time bioluminescence recording of synchronised cells for five consecutive days (representative results in Figure 1A and Figure S1A, respectively). The circadian parameters were obtained directly as an output from the Chronostar software [28], which computes the values for the period, phase, and amplitude based on the raw bioluminescence recordings. To compare the phase between cell lines oscillating with different periods, we converted the phase values to radian. The analysis revealed significant changes in period, phase or amplitude of all KD cells compared to the control cells (Figure 1B). sh*BMAL1* cells show a disrupted pattern of both *BMAL1* and *PER2* promoter activity (Figure 1A and Figure S1A), which agrees with the central role of BMAL1 as a regulator of the core-clock network. Our data showed a significant decrease in the period of oscillations for sh*NR1D1* cells (Figure 1B, T_control_ = 24.9 ± 0.2 h and T_shNR1D1_ = 23.6 ± 0.1 h, *n* = 3, mean ± SEM, *p* < 0.01) and a phase advance for the sh*PER2* cell line (Figure 1B, Phase_control_ = 3.3 ± 0.1 rad and Phase_shPER2_ = 2.9 ± 0.05 rad, *n* = 3, mean ± SEM, *p* < 0.05). We further observed a significant increase in the amplitude for the *PER2* KD cell line (Figure 1B, *p* < 0.01). Furthermore, the antiphase expression pattern of *BMAL1* and *PER2* promoter activity was present in the control, sh*PER2* and sh*NR1D1* cells (Figure 1 and Figure S1).

Also, the relative phase shift between *BMAL1* and *PER2* promoter activity curves, for the same cell line, showed alterations across the KD cells (Figure S1C), for which the *PER2* downregulated cells displayed a significant and larger shift between the peak of *BMAL1* and *PER2*, compared to the control cells (Phase-shift_Control_ = 3.2 ± 0.2 rad and Phase-shift_shPER2_ = 3.6 ± 0.1 rad, *n* = 3, mean ± SEM, *p* < 0.05).

Our gene expression analysis showed significant and specific alterations in the expression of the core-clock genes *PER2*, *CRY1*, *NR1D1*, *CLOCK*, and *BMAL1* in the different KD cell lines compared to the control cell line at 24h after cell synchronisation (Figure 1C). In particular, *BMAL1* downregulation resulted in significant changes in the expression of all core-clock genes analysed, *PER2* and *CRY1* expression were upregulated (*p* < 0.001), while *CLOCK* (*p* < 0.05) and *NR1D1* (*p* < 0.001) were significantly downregulated. Of note, the shRNA-mediated knockdown does not inhibit target gene expression completely, as seen in Figure 1C.

Altogether, these results highlight an important role for the core-clock network in the regulation of the oscillatory phenotype of HCT116 cells, which is likely to have an impact on the expression of target CCGs and alter their controlled cellular functions. 

### 2.2. The Downregulation of Core-Clock Genes Impacts Cell Proliferation, Apoptosis and Migration in CRC Cell Lines 

**Figure 2:**
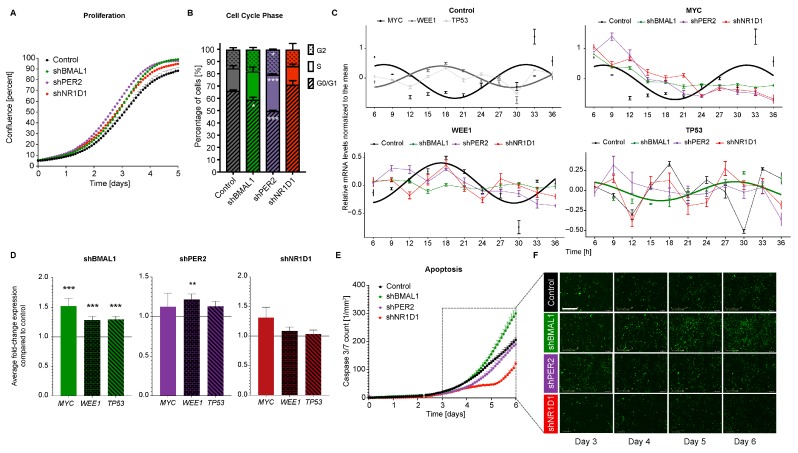
Core-clock gene knockdown affects cell proliferation in HCT116 cells. (**A**) Proliferation analyses of HCT116 cell lines after shRNA) knock-down of core-clock genes (*BMAL1*, *PER2* and *NR1D1*) over 5 days (*n* > 8, mean ± SEM, *p* < 0.001 for *shBMAL1*, *shPER2* and sh*NR1D1* comparing AUC to control, two-tailed unpaired *t*-test). (**B**) Cell cycle phase distribution of KD cell lines compared to control (*n* = 3, mean ± SEM, no asterisk *p* > 0.05, * *p* < 0.05, *** *p* < 0.001; two-tailed unpaired *t*-test). (**C**) 30-hour time-course gene expression analysis for *MYC*, *WEE1* and *TP53* in different HCT116 KD cells (*n* = 3, mean ± SEM, a cosine curve was fitted to all data sets and displayed as a full line for *p* < 0.05, the data points were connected with closed lines if *p* > 0.05). (**D**) The average expression level for *MYC*, *WEE1* and *TP53* in each KD cell line compared to the control (*n* = 3, mean ± SEM, no asterisk *p* > 0.05, * *p* < 0.05, ** *p* < 0.01, *** *p* < 0.001; two-tailed unpaired *t*-test). (**E**) Apoptosis analysis of HCT116 cell lines after shRNA KD of *BMAL1*, *PER2* and *NR1D1* (*n*  > 8, mean  ±  SEM, *p* < 0.05 for *shBMAL1* and *shNR1D1*, two-way ANOVA and corrected for multiple testing using Benjamini and Yekutieli method. Measurements obtained by counting caspase3/7 green objects per mm^2^ every 2 h in the course of 6 days using the IncuCyte S3 device. (**F**) Representation of the apoptosis assay using the IncuCyte S3 software over the course of 6 days. Green dots represent apoptotic cells expressing caspase3/7. Scale bar: 500 µm.

Next, we investigated the putative effect of perturbing the core-clock elements (via the KD of specific core-clock genes) in cellular functioning. For that, we measured proliferation and apoptotic rates of HCT116-KD cells (sh*BMAL1*, sh*PER2* and sh*NR1D1*) compared to the control cell line, using a live-cell analysis system for several days (Figure 2). All HCT116-KD cell lines exhibited significantly higher proliferation rates compared to the control (Figure 2A and Table S1). To evaluate our findings in another CRC cell model, we used SW480 cells, which similar to HCT116 originate from a primary tumour. We measured proliferation in SW480 (SW480-control, SW480-sh*BMAL1*, SW480-sh*PER2*, and SW480-sh*NR1D1*) and also observed an effect of the circadian clock in proliferation, which is very similar to our observations in HCT116-control, HCT116-sh*BMAL1*, HCT116-sh*PER2*, and HCT116-sh*NR1D1* cell lines (Figure S4). These data are consistent with previous studies, which reported enhanced proliferation for cancer cells upon the downregulation of core-clock genes (e.g. *BMAL1* and *PER2*) and highlight a putative role for the clock as tumour suppressor [15,29,30]. Our cell cycle analysis (performed 24 h after cell synchronisation) showed significant changes in the distribution of cell cycle phases for both sh*BMAL1* and sh*PER2* cell lines as compared to the control, with a decreased G0/G1 phase (in both cell lines), as well as increased S and G2 in sh*PER2* cell line (Figure 2B). Additionally, we performed time-course gene expression analysis for key genes involved in the regulation of cell cycle checkpoints (*MYC* and *WEE1*) and of cell apoptosis (*TP53*) (Figure 2C). To evaluate the potential circadian phenotype in the expression of *MYC, WEE1* and *TP53* for that cell line, we compared the normalised time-course expression data with the mean level of expression for each data series and tested if the expression levels oscillate with a period as measured in the bioluminescence recordings of *BMAL1* promoter activity, specific for each cell line. For the sh*BMAL1* cell line with no detectable rhythms of *BMAL1* or *PER2* promoter activity, we tested the existence of oscillations for the different period values obtained from the bioluminescence analysis for the control as well as sh*PER2* and sh*NR1D1* cell lines. Recent studies show that cells with no *Bmal1* expression may have circadian rhythmic expression of other clock-controlled genes [31]. Thus, in our sh*BMAL1* cell line, despite no detectable rhythmic BMAL1-promoter activity, there are still other genes that may show oscillations in expression.

Our time-course data revealed a circadian oscillation (T = 24.9 h, *p* < 0.05) for both *MYC* and *WEE1* expression in the control cells with an antiphase relation between both oscillations. Interestingly, we did not detect significant oscillations of these genes in any of the KD cells, indicating a circadian disruption of gene expression for *MYC* and *WEE1*. *TP53* did not show significant circadian oscillations in the control (T = 24.9 h), as well as *PER2* (T = 24.7 h) and *NR1D1* (T = 23.6 h) downregulated cells, as previously reported for HCT116 WT cells [32] (Figure 2C and Table S2). 

However, we detected significant oscillation of *TP53* in sh*BMAL1* cells for all the tested periods (*p* < 0.05), indicating a differential regulation of *TP53* via *BMAL1* (Figure 2C and Table S2). From the analysis of the average expression of the mentioned cell cycle and apoptosis-related genes for each KD cell line compared to the control cell line, we found *MYC, WEE1* and *TP53* expression to be significantly upregulated in sh*BMAL1* cell line (Figure 2D). Thus, we wondered whether the abnormal expression of *TP53* in sh*BMAL1* cells together with its differential temporal regulation of expression might have an effect on cell apoptosis.

To investigate this hypothesis, we analysed cellular apoptosis by measuring caspase 3/7 fluorescent activity over several days. While sh*BMAL1* cells showed significant higher apoptosis compared to the control, in sh*NR1D1* cells apoptotic rates were significantly decreased (*p* < 0.05, Figure 2E). For the other CRC cells (SW480-control, SW480-sh*BMAL1*, SW480-sh*PER2*, and SW480-sh*NR1D1*), we also observed a dependency of apoptosis on the circadian clock, which is dependent on the particular core-clock gene KD (Figure S4). To better understand the results from the proliferation and apoptosis assays, we quantified the expression levels of *MYC, WEE1* and *TP53* in each HCT116 KD cell line at different time-points compared to the control cell line and analysed the time-course data (Figure S2A and Table S3). This time-course comparison indicated at which time-points the expression of the mentioned genes is higher or lower than the control cell line in each KD condition, which might be useful in effectively targeting gene expression at a specific time-point. Previous studies investigating the role of *BMAL1* overexpression and *NR1D1* activation in HCT116 cells, leading to less cell proliferation and increased apoptosis, respectively, support our findings concerning proliferation and apoptosis of CRC cells [20,29].

**Figure 3:**
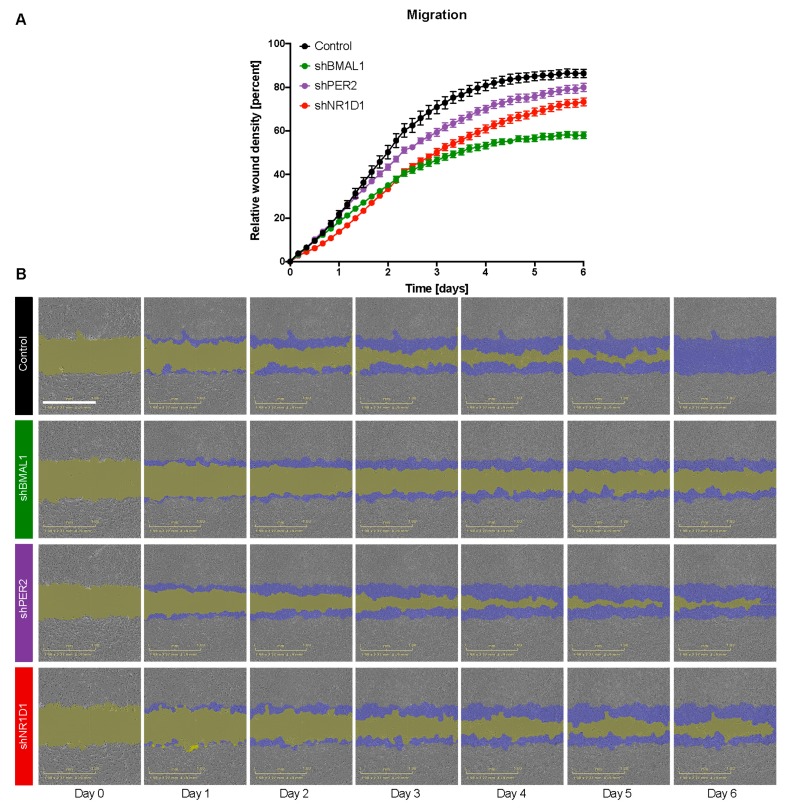
Core-clock gene knockdown affects cell migration in HCT116 cells. (**A**) Migration properties of control and shRNA KD HCT116 cell lines (*shBMAL1*, *shPER2* and *shNR1D1*). Measurements were obtained using a scratch wound assay (IncuCyte). Quantification was performed by measuring the relative wound density over the course of 6 days (*n*  > 5, mean  ±  SEM, *p* < 0.05 for *shBMAL1*, *shPER2* and *shNR1D1* compared to control, two-way ANOVA and corrected for multiple testing using Benjamini and Yekutieli method). (**B**) Representation of the scratch wound assay using the IncuCyte S3 software over the course of 6 days. Blue mask indicates the initial scratch wound area. Gold mask indicates wound border area (cell-free area). Scale bar: 1 mm.

To investigate a putative role for the clock in cell motility, we further examined the effect of perturbing the clock in the migratory behaviour of HCT116-KD cells *in vitro* using a wound-healing assay (Figure 3). Our data showed a significant decrease in migration activity of HCT116-KD cells as compared to the control cell line (*p* < 0.05). In particular, sh*BMAL1* cells show the least migration followed by sh*NR1D1* and sh*PER2* cells (Figure 3A). For the SW480 cell line, sh*BMAL1* and sh*NR1D1* cells also showed decreased migration compared to the control (Figure S4). In contrast, we did not observe a significant different migration behaviour in SW480 sh*PER2* cells (Figure S4).

In summary, our *in vitro* analyses of CRC downregulated cells indicate a prominent role of circadian core-clock genes in mediating vital cellular properties such as proliferation, apoptosis and cell migration. 

### 2.3. Downregulation of BMAL1 and NR1D1 Impairs Tumorigenesis In Vivo

**Figure 4:**
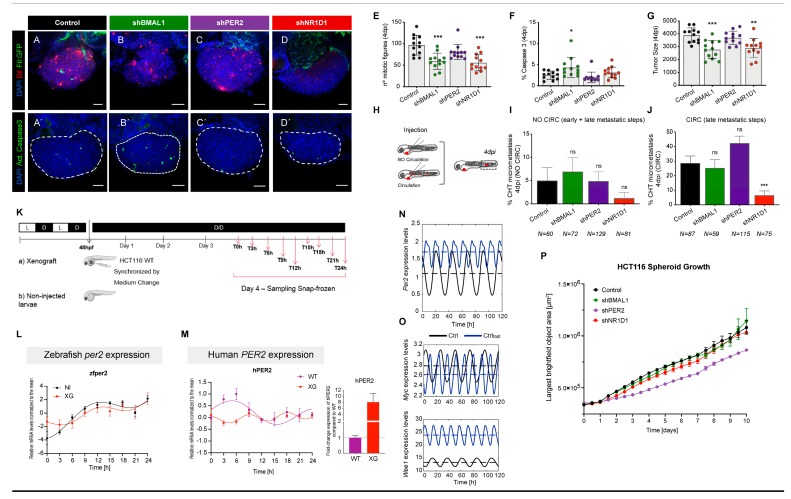
(**A**–**D**)—Representative confocal images of zebrafish larvae xenografts. Human CRC HCT116 control and KD cells (*shPER2*, *shBMAL1* and *shNR1D1*) were labelled with DiI dye (red) and injected into the PVS of 2dpf Tg(Fli:eGFP) zebrafish (*n* = 12). At 4dpi mitotic figures (**E**), % of apoptosis (**F**—Activated caspase 3), tumour size (**G**—number of tumour cells) and metastatic potential (**H–J**) were quantified. Metastatic potential was quantified by injecting cells into the Perivitelline Space (PVS) only, with no cells in circulation (**I**) and cells injected directly into circulation (**J**). All images show xenografts anterior to the left, posterior to the right, dorsal up and ventral down. Each dot represents one xenograft. Results are from one single independent experiment. Statistical analysis was performed using Mann–Whitney test (ns or no asterisk *p* > 0.05, * *p* ≤ 0.05, ** *p* ≤ 0.01, *** *p* ≤ 0.001). Error bars: mean ± SEM. Scale bars: 50µm. (**K**) Overview of the time-course for the *in vivo* study. HCT116 cells were injected into zebrafish larvae at 48h post-fertilisation (48 hpf). After 3 days, xenograft larvae (XG) were collected every 3h for the course of 24h during dark/dark conditions. Non-injected zebrafish larvae were used as controls (NI), *n* = 45 larvae/time-point. Time-course mRNA expression levels compared to the mean for: (**L**) zebrafish *per2* gene in non-injected zebrafish larvae (black line and dots) and zebrafish xenografts injected with HCT116 cells (red line and dots), (**M**) human *PER2* gene in zebrafish xenografts injected with HCT116 cells (red line and dots) and in human in HCT116 WT cells (purple line and dots). The average h*PER2* expression between XG and HCT116 WT cells is depicted as a bar plot. Cosinor fitting curves were applied for the determination of oscillation parameters (Table S5, Table S7 for list of primers for RT-qPCR, no asterisk *p* > 0.05). (**N–O**) Shown are the *in silico* expression profiles for (**N**) PER2 (**O**) MYC and WEE1 in Ctrl (T = 23.7 h, black) and Ctrlfast conditions (T = 13.8 h, blue). (**P**) HCT116 spheroid growth rates over time upon core-clock gene knockdown (*shBMAL1*, *shPER2* and *shNR1D1*) compared to the control. *n* = 5, mean  ±  SEM, *p* < 0.05 for *shPER2* compared to control using two-way ANOVA and corrected for multiple testing using Benjamini and Yekutieli method.

To test *in vivo* the impact on tumorigenesis resulting from the downregulation of the core-clock genes *BMAL1*, *PER2*, and *NR1D1*, we used the zebrafish larvae (*Danio rerio*) xenograft model [24]. HCT116 cells transduced with control, sh*BMAL1*, sh*PER2*, and sh*N1RD1* constructs were injected into 2 days post-fertilisation zebrafish embryos. At 4 days post-injection (4 dpi), we analysed the impact of the downregulation of clock genes on the previously studied hallmarks of cancer: proliferation (quantification of mitotic figures), apoptosis (quantification of Activated Caspase3), tumour size and metastatic potential (Figure 4A–J). 

To assess the metastatic potential of each tumour, we quantified the capacity of tumour cells to form micrometastasis in the caudal hematopoietic tissue (CHT) located in the tail region, the most distant site from injection [24] (Figure 4H–J). In our zebrafish model, we injected the CRC cells in two different ways: in the Perivitelline Space (PVS) only, with no cells in circulation (Figure 4I, similar to subcutaneous injection in the mouse) or directly into circulation (Figure 4J, similar to experimental metastasis in the mouse). In the first scenario, for tumour cells to efficiently establish a micrometastasis, they must go through all the metastatic steps (from early to late steps) i.e. invade neighbouring tissues, intravasate into a blood vessel, survive circulation and extravasate to colonise a distant site. In contrast, in the second scenario i.e. the “experimental metastasis”, cells only need to survive circulation, extravasate and colonise, i.e. the late metastatic steps. Therefore, although in our short assay we were not evaluating an “evolutionary metastasis”, we could evaluate the metastatic potential, i.e. the ability that these cells have to undergo these different behaviours.

Our results showed that *BMAL1* KD led to a significant reduction in proliferation (~40% reduction, *p* = 0.0007), induction of apoptosis (1.4-fold induction, *p* = 0.0152) accompanied by a reduction of tumour size (30% reduction, *p* = 0.0003). However, we could not detect a significant impact on the metastatic potential (Figure 4I,J). In contrast, KD of *PER2* had no significant impact on proliferation, apoptosis or micrometastasis frequency (Figure 4E–J). On the other hand, and similar to *BMAL1* KD, the downregulation of *NR1D1* led to a significant reduction of proliferation (~50% reduction, *p* = 0.0008) and tumour size (~24% reduction, *p* = 0.0012) (Figure 4E–G). Moreover, we observed that when cells were injected directly into circulation (CIRC), *NR1D1* KD reduced the capacity of the cells to form micrometastasis in the tail region by 75% (Figure 4J, from ~29% in control, *n* = 87 to ~7% in *NR1D1* KD, *n* = 75, *p* = 0.0004 Fisher’s exact test). Hence, we observed a reduction of the number of xenografts with micrometastasis by the end of the assay. However, when cells were not injected into circulation this phenotype was not so clear (Figure 4I), suggesting that *NR1D1* might have a role in the late metastatic steps. The average and total number of cancer cells in each condition at 4dpi, as well as the raw data regarding the micrometastasis assay are provided in Table S4.

Unexpectedly, in contrast to our *in vitro* results where we observed an increase in cell proliferation as a consequence of the KD of core-clock genes, we now measured an overall decrease in cell proliferation (number of mitotic figures) and a consistent decrease in the tumour size in the *in vivo* zebrafish xenograft model. We wondered if the zebrafish host could affect the HCT116 xenotransplant and give rise to a different clock as compared to the same cells *in vitro*. To test this, we injected HCT116 cells into zebrafish embryo at 48 h post-fertilisation (48 hpf) and after 3 days collected xenograft zebrafish larvae (XG) every 3h for the course of 24h during dark/dark conditions (Figure 4K), to avoid the possible influence of light on the zebrafish circadian clock, since all peripheral tissues of zebrafish can be synchronized with light directly and independently [33]. Non-injected zebrafish embryos were used as controls (NI). Given the high sequence similarity for core-clock genes between both species, we selected for the time course analysis the zebrafish gene *per2* since the sequence identity is the lowest (48%), compared to other core-clock genes (*Bmal1* = 85%, *Cry1* = 79%, *Clock* = 70%, *Nr1d2* = 65% and *Rorα* = 91%). We carried out an RT-qPCR quantification of the time-course mRNA expression levels compared to the mean for: (a) zebrafish *per2* gene in non-injected zebrafish larvae and zebrafish xenografts injected with HCT116 WT cells (Figure 4L), (b) human *PER2* gene in zebrafish xenografts injected with HCT116 cells and (c) HCT116 WT cells *in vitro* (Figure 4M). Interestingly, xenotransplanted HCT116 cells showed a *PER2* expression profile with a reduced period as compared to *in vitro* cells (T_HCT116_WT_ = 20 ± 1.6 h and T_HCT116_XG_ = 9 ± 2.1 h, Figure 4M and Table S5). Additionally, the average expression of human *PER2* in the xenograft was higher than the HCT116 WT cells outside the larvae (Figure 4M). Our result suggests that the reduced xenograft *PER2* period could eventually lead to phenotypic differences between *in vitro* and *in vivo* HCT116 in terms of cell cycle progression and apoptosis. 

We further explored this idea using a mathematical model for the core-clock [17] to simulate a scenario with a faster period (T_fast_ = 13.8 h, vs. Ctrl condition with T = 23.7 h) for the *PER2* gene (Figure 4N). Our aim was to do a qualitative study regarding the impact of a shorter *Per2* period (by simulating a reduction of about 10h for the period of *Per2*, as observed experimentally) in the mean values of cell cycle genes, rather than to reproduce the exact period values observed experimentally. We generated 1000 different sets of model parameters by randomly changing the parameter values between −80% and +80% of the original values. To simulate T_fast_ we selected the parameter set that yielded the lowest period of *Per2*. To avoid overfitting, when generating the different sets of model parameters, we did not constrain the mean value of *Per2* to remain in the same value as with the original parameters. Hence, the mean value of *Per2* was allowed to change in every random simulation as a result of the model parameters (Table S6). This perturbation had an impact on the average gene expression of *Myc* and *Wee1* (Figure 4O). We observed that the decrement on the period of *PER2* led to an upregulation of this gene (Figure 4N), accompanied by a downregulation of *MYC* and an upregulation of *WEE1*—pointing to reduced proliferation. These results suggest that the zebrafish host is able to modify the proliferative capacity of HCT116 cells. The impact that we observe in *Myc* and *Wee1* cannot be attributed uniquely to the faster period, but could also be due to the altered mean expression of *Per2* in the fast condition or other model outputs, which were not forced to remain equal to the control scenario, but that have nevertheless changed upon the forced perturbation in the period of *Per2*.

It is also possible that the tumour architecture for cells growing inside the host in a 3D environment may influence proliferation, as compared to the *in vitro* 2D cell culture model. Our *in vitro* data using HCT116 cells grown as 3D spheroids showed an overall reduced size for the cell lines carrying a stable knockdown of *BMAL1*, *PER2* and *NR1D1* compared to the control cell line (*p* < 0.05 for sh*PER2*, Figure 4P). 

Altogether, our results suggest an essential role for *BMAL1* and *NR1D1* in survival of HCT116 tumours and point to an *NR1D1* specific role in metastatic potential *in vivo*.

## 3. Discussion

The circadian clock affects numerous aspects of physiology and its correct working is vital for the proper functioning of organismal and cellular homeostasis. Thus, malfunctions of the circadian clock may directly or indirectly trigger abnormal cellular processes, eventually leading to the onset, or further progression, of cancer. Several studies point to a bidirectional interplay between circadian regulation and cancer with implications in therapy response (reviewed in [2,9]). 

In this work, using *in vitro* CRC cell lines and an *in vivo* xenograft model, we report how the disruption of the circadian clock via the downregulation of the core-clock genes *BMAL1*, *PER2* and *NR1D1* impact proliferation, apoptosis, migration, and invasion. 

Our *in vitro* results indicate a global change in the expression of several core-clock genes in the knockdown cells with subsequent significant alterations in their clock phenotypes (Figure 1 and Figure S1). These changes were more prominent upon the downregulation of *BMAL1* in HCT116 cells, highlighting the central role of this gene in maintaining the clock functionality. Accordingly, the reduction of *BMAL1* expression led to an upregulation of *MYC* and *TP53* expression, as well as of genes involved in cell cycle regulation (e.g. *CDK4*, *CDK2* and *CCND1*), resulting in higher proliferation and apoptotic activities as compared to the other knockdown conditions (Figure 2, Figure S2A and Figure S3A). These findings corroborate previously reported impact of *BMAL1* in mediating proliferation in HCT116 cells (higher and lower proliferation rates upon *BMAL1* silencing and overexpression, respectively) [29] and the inhibitory role of CLOCK/BMAL1 on *MYC* expression [34]. Furthermore, we observed an inhibiting effect of *BMAL1* on apoptosis using both *in vitro* and zebrafish *in vivo* experiments (Figure 2E and Figure 4F), which is accompanied by altered levels of *TP53* and its differential circadian regulation via *BMAL1*. *NR1D1* downregulation, however, resulted in reduced cellular apoptosis, which may result from the elevated *BMAL1* levels induced by the KD in these cells and is further reinforced by the apoptosis-inducing effect of *NR1D1* activation reported in HCT116 cells [20]. 

Another important hallmark of cancer is the ability of cells to migrate and undergo several steps in order to metastasize. To investigate this, we performed *in vitro* migration assays, as well as *in vivo* zebrafish experiments to quantify the metastatic potential of HCT116 cells carrying stable knockdowns of core-clock genes as mentioned above. For this, we introduced tumour cells in the PVS and the spread of these cells requires the intravasation from the primary site of injection followed by blood survival and, finally, extravasation to the surrounding tissues in the caudal region. Using this procedure, we aimed at mimicking the several stages (but not all) of the metastatic process. Both our *in vitro* and *in vivo* results suggest an impairment of cell motility and a 20% reduction in metastatic potential upon *NR1D1* knockdown in HCT116 cells (Figure 3 and Figure 4J). We observed as well very similar effects *in vitro* for SW480-sh*BMAL1*, SW480-sh*PER2* and SW480-sh*NR1D1* cells regarding proliferation and migration (Figure S4). These findings were supported by our gene expression analysis on EMT and CRC stem cell marker genes in downregulated *NR1D1* cells, where the expression of *SNAI1* and *CD44* were significantly downregulated compared to the control cells (Figure S3B). 

Consistent with our *in vitro* results, the *in vivo* zebrafish xenograft model showed similar and enhanced apoptosis rates for *BMAL1* knockdown cells compared to the *in vitro* data, as well as reduced migration/metastatic potential for *NR1D1* downregulated cells. However, we observed a differential effect when comparing cell proliferation *in vitro* and tumour growth *in vivo*. We speculated, that the xenotransplantation of HCT116 cells in zebrafish larvae could impact the proliferative abilities of the cancer cells possibly via the circadian clock. To investigate this hypothesis, we conducted a time-course gene expression analysis for *PER2* (the clock gene with the least sequence similarity between the two organisms) using zebrafish xenografts compared to non-injected embryo and HCT116 cells cultured *in vitro*. We observed a remarkable change in the oscillation pattern and the level of human *PER2* expression in WT HCT116 cells between the *in vivo* and *in vitro* assays (Figure 4M and Table S5) pointing to an influence on the HCT116 clock via the host. We then applied a mathematical model to predict the theoretical outcome of an alteration of the *PER2* oscillatory phenotype on the expression profile of the cell cycle inhibitor *Wee1* and the proto-oncogene *Myc* (Figure 4N,O). Our simulations showed a general increase in *Wee1* and a decrease in *Myc* levels, which would explain an overall less proliferation of cancer cells inside the zebrafish. In addition to the modelling approach, the data from our spheroid assay indicates that the 3D architecture of cells in the host may have an impact on the reduced cell proliferation observed *in vivo* and offer an alternative explanation for the observed phenotype (Figure 4P). Altogether, our data highlights a profound role for tumour microenvironment in affecting cancer behaviour in terms of proliferation, apoptosis and metastatic potential, likely due to the cancer/host circadian clock interplay, as seen previously using co-culture experiments with colon cancer cells [35]. Our work highlights the different functionalities of the colon cancer circadian clock as a function of their environmental conditions (*in vitro* vs. *in vivo*), and their implications on the cancer phenotype. Future work will be necessary to fully address these interesting open questions.

## 4. Materials and Methods

### 4.1. Cell Culture

HCT116 cells (ATCC^®^ CCL-247™) and SW480 cells (RRID:CVCL_0546) were cultured in Dulbecco’s Modified Eagle Medium DMEM (Gibco, Thermo Fisher Scientific, Waltham, MA, USA) supplemented with 10% FBS (Gibco, Thermo Fisher Scientific, Waltham, MA, USA) and 1% Penicillin−Streptomycin (Gibco, Thermo Fisher Scientific, Waltham, MA, USA) in a humidified atmosphere containing 5% CO_2_ at 37 °C. Stable-transduced cells were selected and maintained in medium containing 150 μg/mL hygromycin B (Gibco, Thermo Fisher Scientific, Waltham, MA, USA) for the BMAL1:LUC hygromycin (BLH), 10 μg/mL of blasticidin S HCl (Gibco, Thermo Fisher Scientific, Waltham, MA, USA) for the PER2:LUC blasticidin (PLB) and 1.5 μg/mL of puromycin (Gibco, Thermo Fisher Scientific, Waltham, MA, USA) for the shRNA KD of the clock genes. For live-cell bioluminescence recording, cells were maintained in phenol red-free DMEM (Gibco, Thermo Fisher Scientific, Waltham, MA, USA) containing 10% FBS, 1% Penicillin−Streptomycin and 250µM D-Luciferin (Bio-Rad laboratories, Hercules, CA, USA). Cell counting and morphology analysis were performed in LUNA™ Automated Cell Counter (Logos Biosystems, Anyang, South Korea). Cell lines were tested for mycoplasma by using the Mycoplasmacheck service of Eurofins Genomics (Eurofins Genomics, Ebersberg, Germany).

### 4.2. Lentivirus Production

Lentiviral elements containing a *BMAL1*-promoter-driven luciferase (BLH), a *PER2*-promoter-driver luciferase (PLB), an empty vector (TRC Lentiviral pLKO.1 Empty Vector Control; Dharmacon Inc., Lafayette, CO, USA) or shRNA KD (TRC Lentiviral Human ARNTL shRNA—Clone ID: TRCN0000019096/97; TRC Lentiviral Human PER2 shRNA—Clone ID: TRCN0000018542; TRC Lentiviral Human NR1D1 shRNA—Clone ID: TRCN0000022174; Dharmacon Inc., CO, USA) were used in this work. For lentivirus production, HEK293T (human, kidney, ATCC Number: CRL-11268) cells were seeded in 175cm culture flasks and co-transfected with 12.5 μg packaging plasmid psPAX, 7.5 μg envelope plasmid pMD2G and 17.5 μg expression plasmid (BLH, PLB or shRNA KD) using the CalPhos mammalian transfection kit (Clontech, Mountain View, CA, USA) according to the manufacturer’s instruction. To harvest the lentiviral particles, the supernatant was centrifuged at 4100× *g* for 15 min to remove cell debris and passed through a 45 μm filter (Sarstedt, Nümbrecht, Germany). The lentiviral particles were stored at −80 °C.

### 4.3. Transduction with Lentiviral Vectors

For lentiviral transduction, 1 × 10^5^ cells were seeded in 6-well plates. On the day of transduction, 1.5 mL of supernatant of the corresponding lentivirus were added to each well. 8 μg/mL protamine sulfate (Sigma-Aldrich, St. Louis, MO, USA) and 4 μg/mL polybrene (Sigma-Aldrich, St. Louis, MO, USA) was used to enhance transduction efficiency. After 48h, the medium was replaced and selection medium was added (complete growth medium containing appropriate antibiotic) to obtain stably transduced cells and incubated at 37 °C with 5% CO2 atmosphere. Untransduced cells treated with the same antibiotic concentration were used as selection controls.

### 4.4. Bioluminescence Measurements

For live-cell bioluminescence recordings, 2.5 × 10^5^ HCT116 cells were seeded in 35 mm dishes and maintained in phenol red-free DMEM (Gibco, Thermo Fisher Scientific, Waltham, MA, USA) containing 10% FBS, 1% Penicillin-Streptomycin supplemented with 250 µM D-Luciferin (Bio-Rad laboratories, Hercules, CA, USA). Cells were synchronized by adding fresh medium prior to measurement (zeitgeber time = 0 h). *BMAL1*-promoter-(BLH)-reporter or *PER2*-promoter-(PLB)-reporter activities were measured, using a LumiCycle instrument (Actimetrics, Wilmette, IL, USA) for five consecutive days. Raw luminescence data were de-trended by the 24 h running average (divided values) using the Chronostar analysis software V3.0 (https://www.achim-kramer-lab.de/downloads/chronostar.exe) [28]. The first 12 h of measurement were removed from the analysis, since the first data collection is comparatively very noisy due to technical limitations of the device. The phase in radian was calculated using the following equation:

$$\varphi \left(rad\right)=\varphi \left(h\right)\cdot \left(\frac{2\cdot \pi }{T}\right)$$

with φ(h) = phase (in h), *T* = period 

### 4.5. Animal Care and Handling

Zebrafish (*Danio rerio*) Tg(fli1:eGFP) were handled and maintained according to the standard protocols of the European Animal Welfare Legislation, Directive 2010/63/EU (European Commission, 2016) and Champalimaud Fish Platform. The study protocol was approved by the Portuguese institutional organisations ORBEA (Órgão de Bem-Estar e Ética Animal/Animal Welfare and Ethics Body) and DGAV (Direção Geral de Alimentação e Veterinária/Directorate General for Food and Veterinary) 2015/005.

### 4.6. Cell Labelling

Before injection, tumour cells were labelled in flask with vybrant CM-DiI (Life Technologies, Eugene, USA) at 4 µL/mL concentration, diluted in sterile PBS 1X (Biowest, Riverside, MO, USA) for 10 min at 37 °C, according to supplier guidelines. Cells were then resuspended in sterile PBS to a final concentration of 0.25 × 10^6^ cells/µL.

### 4.7. Zebrafish Xenograft Microinjection

Zebrafish with 2 days post-fertilisation (2 dpf) were anesthetized by immersion in Tricaine MS-222 (Sigma, Merck, St Louis, MO, USA). Approximately 1500 labelled cells were injected into the periviteline space (PVS) of zebrafish under a fluorescence scope with a mechanical micropipetor attached (World Precision Instruments, Pneumatic Pico Pump PV820, Sarasota, FL, USA). After injection, zebrafish larvae remained for 15 min in Tricaine and then were transferred to E3 medium and kept at 34 °C in the dark, until the end of the experiment (4 days). The zebrafish larvae were kept under dark/dark conditions after microinjection in order to avoid the influence of the light on the circadian clock of the larvae, since the clock of zebrafish is directly entrained by light [33]. 

At 1 day post-injection (1dpi), zebrafish xenografts were screened according to the presence or absence of tumour mass and grouped according to its tumour size. Four days later, zebrafish xenografts were sacrificed with Tricaine and fixed with 4% Formaldehyde (Life Technologies, Eugene, USA) and preserved at −20 °C in 100% methanol to further analysis. 

### 4.8. Micrometastasis Assay

To assess the metastatic potential of each tumour, we quantified the capacity of tumour cells to form micrometastasis in the caudal hematopoietic tissue (CHT) located in the tail region. To distinguish between early vs. late stages of the metastatic cascade we compared the micrometastasis efficiency when cells were placed directly into circulation vs. when not. For that, we injected the cells into the PVS alone or directly into circulation (Figure 4H). At 1hpi all xenografts were re-screened to confirm that no cells have escaped into circulation due to injection. At 4dpi, we analysed the number of xenografts that presented micrometastasis away from the PVS injection site i.e. in the CHT. 

### 4.9. Immunofluorescence

Whole-mount immunofluorescence was performed as previously described [24]. Primary antibody: anti-activated caspase-3 (rabbit, Cell Signaling, # 9661, 1:100) and secondary antibody anti-rabbit 647 (1:400). Nuclei were stained with DAPI (blue) and xenografts mounted in between 2 coverslips, allowing double side acquisition using Mowiol mounting media (Sigma, Merck, St Louis, MO, USA).

### 4.10. Imaging Processing and Analysis

The z-stacks acquisitions were acquired with a Zeiss LSM 710 confocal microscope with 5µm interval, with an average of 15 slices in total. Generated images were analysed using the FIJI/ImageJ software and cell counter plugin. Tumour size is the average number of cells (DAPI) of three slices (Zfirst—first tumour slice, Zmiddle—tumour slice, Zlast—last slice of the tumour) x total slices number/1.5. Mitosis and apoptotic cells (activated caspase 3 positive cells) were quantified in all tumour slices.

### 4.11. RNA Extraction, cDNA Synthesis (Reverse Transcription) and Quantitative Real-Time PCR (qPCR)

Total RNA was isolated using the RNeasy Plus Mini kit (Qiagen, Hilden, Germany) according to the manufacturer’s manual. Prior to the purification procedure, medium was discarded and cells were washed with PBS and lysed in RLT Plus buffer (Qiagen, Hilden, Germany). Genomic DNA was digested using gDNA eliminator columns provided with the kit (Qiagen, Hilden, Germany). RNA was eluted in 25–50 µL RNase-free water. The final RNA concentration was measured using a Nanodrop 1000 (Thermo Fisher Scientific, Waltham, MA, USA). RNA was then stored at −80 °C until use. RNA extraction from zebrafish larvae was performed using TRIzol reagent (Invitrogen, Thermo Fisher Scientific, Carlsbad, CA, USA) and by following the manufacturer’s protocol. The aqueous phase contains the RNA was mixed with 70% ethanol and transferred into a RNeasy spin column (Qiagen, Hilden, Germany) followed by washing and purifications steps as instructed by the manufacturer (RNeasy Mini Kit, Qiagen, Hilden, Germany). 1 µg of total RNA was reverse-transcribed to cDNA with M-MLV reverse transcriptase (Invitrogen, Thermo Fisher Scientific, Carlsbad, CA, USA), random hexamers (Thermo Fisher Scientific, Waltham, MA, USA) and dNTPs Mix (Thermo Fisher Scientific, Waltham, MA, USA). RT-qPCR was performed using human QuantiTect Primer assays (Qiagen, Hilden, Germany) and SsoAdvanced Universal SYBR Green Supermix (Bio-Rad laboratories, Hercules, CA, USA) in 96-well plates. *GAPDH* was used as reference genes. The following primers were used for zebrafish gene expression analysis: *per2* (fw: 5′-ATGTCGATGGCTTTAGGCAG-3′; rev: 5′-CGAGACATCCAGAAGGTGCT-3′) and *gapdh* (Qiagen, Hilden, Germany). A list of all other primers designed in-house including their sequence is provided in Table S7. These genes were selected due to their low conservation in both organisms to avoid cross-species activity in later experiments in *Danio rerio* xenografts. The qPCR reaction and the subsequent melting curve were performed using a CFX Connect Real-Time PCR Detection System (Bio-Rad laboratories, Hercules, CA, USA). A melting curve analysis was performed to detect potential unspecific amplification products. Cq values were determined using the regression method. The expression levels were normalised to those of *GAPDH* (^Δ^CT) and calibrated to the mean expression value of each gene (time-course analysis) or in relation to the respective control (^ΔΔ^CT). Relative quantification was calculated using the 2^−ΔΔCt^ method. Biological and technical replicates were included in the analysis. The mean and the standard error of the mean were calculated.

### 4.12. Cell Cycle Assay

1 × 10^6^ cells under the logarithmic phase were collected 24 h after synchronisation, washed with 1× PBS (Gibco, Thermo Fisher Scientific, Waltham, MA, USA) and fixed with ice cold 80% ethanol. Subsequently samples were washed with PBS and incubated in a 200 µL of PBS solution containing 0.5% Tween20 (Sigma, Merck, St Louis, MO, USA), 1% BSA (Sigma, Merck, St Louis, MO, USA), 2 N HCl/Triton X-100 (Sigma, Merck, St Louis, MO, USA) and 10 mg/mL of RNase (AppliChem, Cat. no. A2760) for 30 minutes at room temperature. For PI staining, the supernatant was removed after a centrifugation, the fixed cell pellets were resuspended and stained in 500 μL of PBS containing 50 μM PI (Sigma, Merck, St Louis, MO, USA) for 30 minutes at 37 °C. Subsequently, the supernatant containing PI solution was removed and the stained cells were resuspended in 500 µL PBS and read in FACS Cabilur (Becton Dickinson, Franklin Lakes, NJ, USA). The cell cycle analysis was conducted by fitting a univariate cell cycle model using the Watson pragmatic algorithm as implemented in FlowJo v10.2 (FlowJo LLC, Ashland, OR, USA, https://www.flowjo.com/solutions/flowjo/downloads).

### 4.13. Proliferation, Apoptosis, and Migration Measurements

#### 4.13.1. Proliferation Assays

For proliferation assay, 5000 cells/well (HCT116) or 10,000 cells/well (SW480) were seeded in a 96-well plate (Sarstedt, Nümbrecht, Germany). Cells were allowed to adhere and placed in the IncuCyte^®^ S3 Live Cell System Analysis (Sartorius, Göttingen, Germany). Four pictures were recorded every two hours for biological and technical replicates. Analysis were performed by using IncuCyte S3 Software (Sartorius, Göttingen, Germany).

#### 4.13.2. Apoptosis Assays

HCT116 cell lines (sh*BMAL1*, sh*PER2* and sh*NR1D1*) as well as empty-vector control cells were seeded in 6-well plates containing complete media. At around 80% confluence, cells were washed once with 1× PBS (Gibco, Thermo Fisher Scientific, Waltham, MA, USA) and counted using a Luna Automated Cell Counter. Cells were seeded in a 96-well plate (Sarstedt, Nümbrecht, Germany) at a concentration of 5000 cells/100 µL medium (HCT116) or 10000cells/100 µL medium (SW480) and incubated for 24 h in an incubator at 37 °C with 5% CO_2_. For each cell line biological replicates and technical replicates were prepared. After 24 h incubation, cell media were replaced with fresh medium containing caspase 3/7 (Sartorius, Göttingen, Germany, 1:2000. Cell apoptosis was measured using the IncuCyte^®^ S3 Live Cell System Analysis. Cells were scanned every 3 h with a 10× objective and by using the phase and green image channels.

#### 4.13.3. Migration Assays

For the migration assay, 35000 cells/well (HCT116) or 40000 cells/well (SW480) were seeded in a 96-well Essen ImageLock^TM^ microplate (Sartorius, Göttingen, Germany) and incubated overnight at 37 °C, 5% CO_2_. In the next day, the WoundMaker^TM^ (Sartorius, Göttingen, Germany) was used to create precise and reproducible wounds. The medium was replaced with fresh medium and the plate was placed in the IncuCyte^®^ S3 Live Cell System Analysis (Sartorius, Göttingen, Germany). Image acquisition was performed by setting the “scan type” to Scratch Wound and Wide Mode, using the 10× objective. Plate was scanned every hour. Analysis was performed with the scratch wound method in the IncuCyte S3 Software (Sartorius, Göttingen, Germany).

#### 4.13.4. 3D Spheroid Assay

HCT116 cells (100 µL, 5000 cells/well) were seeded in 96-well ultra-low attachment plates (S-BIO, Hudson, NH, USA) in growth medium. The plate was centrifuged at 125× *g* for 10 min at room temperature in order to initiate spheroid formation. The cells were then monitored every day using IncuCyte live-cell analysis system (Sartorius, Göttingen, Germany). Once spheroids reach the desired size (between 200–500 µm) and after three days, cell growth was monitored for an additional 10 days using IncuCyte Spheroid Software Module. Spheroid growth quantification was performed using largest brightfield object mask. 

### 4.14. Rhythmicity Analysis of Time-Course Data

#### 4.14.1. Harmonic Regression Using R

Circadian parameters were identified for RT-qPCR results by fitting a linear sine-cosine function using the Harmonic Regression package in R software [36]. The analysis was performed for periods between 23–25 h (range of the bioluminescence data) with a sampling interval of 0.1. Harmonic regression fits a model to time-series using y(t) = m+ a × sin (2 × π × t/ω) + b × cos (2 × π × t/ω). Estimated amplitudes (A= √ (a2 + b2)) and phases (tan φ = b/a) along with *p*-values (according to F-test) and Benjamini-Hochberg adjusted *p*-values (*q*-values) can be found in the Table S2 and Table S3.

#### 4.14.2. Cosinor Analysis

Cosinor analysis of circadian oscillation of in vivo time-series data was performed using the Prism software with the following equation for non-linear regression of time-series data to a cosine curve:

$$F\left(t\right)=M+A\cdot (\cos\frac{2\cdot \pi \cdot t}{T}+\varphi )+S\cdot t$$

with *M* = mesor, *A* = amplitude, *T* = period, *φ* = acrophase, *S* = slope, *t* = time.

### 4.15. Statistical Analysis

All data is presented as mean ± SEM. * *p* < 0.05, ** *p* < 0.01, *** *p* < 0.001. Statistical analysis was performed using Prism software. Cell proliferation was analysed by comparing area under the curve (AUC) data between the control and KD conditions and tested using two-tailed unpaired *t*-test. All data sets were challenged by a normality test. Data sets with a Gaussian distribution were analysed by unpaired *t*-test. Data sets that did not pass the normality test were analysed by Mann Whitney test. 

### 4.16. Mathematical Modelling

We used a published model by our group which mimics the interplay between the core-clock machinery and cell cycle components [17] to simulate a reduction on the period of *PER2*, as observed in the xenotransplantation of Ctrl HCT116 cells. The model output with the original set of parameters [17] was considered as the *in silico* Ctrl scenario. We then generated 1000 different sets of model parameters by randomly changing the parameter values between −80% and +80% of the original value. To avoid parameter sets producing null oscillations or producing oscillations with very large periods, we imposed boundaries for the parameter set as (1) an amplitude of the Per gene larger than 10–5 a.u, and (2) a period of *PER2* < 40 h. The original parameter values used to simulate the Ctrl scenario are specified in the original publication [17]. In order to simulate a condition with shorter period of Per gene as compared to Ctrl (Ctrl_fast_), we selected the parameter set leading to the lowest possible period of Per gene (13.8 h). The parameter values that were modified from the original publication [17] and used to simulate the Ctrl_fast_ (Figure 4N,O) condition are provided in Table S6. 

## 5. Conclusions

In summary, the present study shows that circadian core-clock genes are involved in key cancer properties in colorectal cancer cell lines and proposes the regulation of cell migration, invasion, and metastatic potential by *NR1D1* in zebrafish xenografts.

## Supplementary Materials

The following are available online at https://www.mdpi.com/2072-6694/12/4/853/s1, Figure S1: *PER2* promoter activity show different oscillation patterns in HCT116 knockdown cell lines. Figure S2: 30-hour time-course gene expression analysis for *MYC*, *WEE1* and *TP53* in different HCT116 KD cells. Figure S3: Gene expression analysis of cell cycle-related genes and genes involved in EMT. Figure S4: Core-clock gene knockdown alters cellular properties of SW480 colon cancer cell lines. Table S1: AUC analysis of proliferation data of HCT116 control and KD cell lines depicted in Figure 2A. Table S2: Summary of the fitting parameters (period, phase, amplitude) for the different time-course experiments depicted partially in Figure 2C. Table S3: Summary of the fitting parameters (period, phase, amplitude and mesor) for the different time-course experiments depicted in Figure S2A. Table S4: Number of cancer cells for each experimental group for the *in vivo* study (Figure 4E–G) as well as raw data for the analysis of metastatic potential (Figure 4H–J). Table S5: Summary of the fitting parameters (period and phase) for the different time-course experiments depicted in Figure 4L,M. Table S6: Model parameters used to simulate Ctrl_fast_ condition (Figure 4N,O). Table S7: List and sequence of all primers designed in-house which were used for RT-qPCR analysis.
