# Supplementary material for: The Core-Clock Gene NR1D1 Impacts Cell Motility In Vitro and Invasiveness in a Zebrafish Xenograft Colon Cancer Model
Source: Cancers (Basel). 2020 Apr 1;12(4):853. doi: 10.3390/cancers12040853 (PMC7226575; doi:10.3390/cancers12040853)
Supplement: Supplementary file 1 [file cancers-12-00853-s001.zip › Supplementary_Material/Supplementary_Figures.docx]

**SUPPLEMENTARY MATERIALS**

***
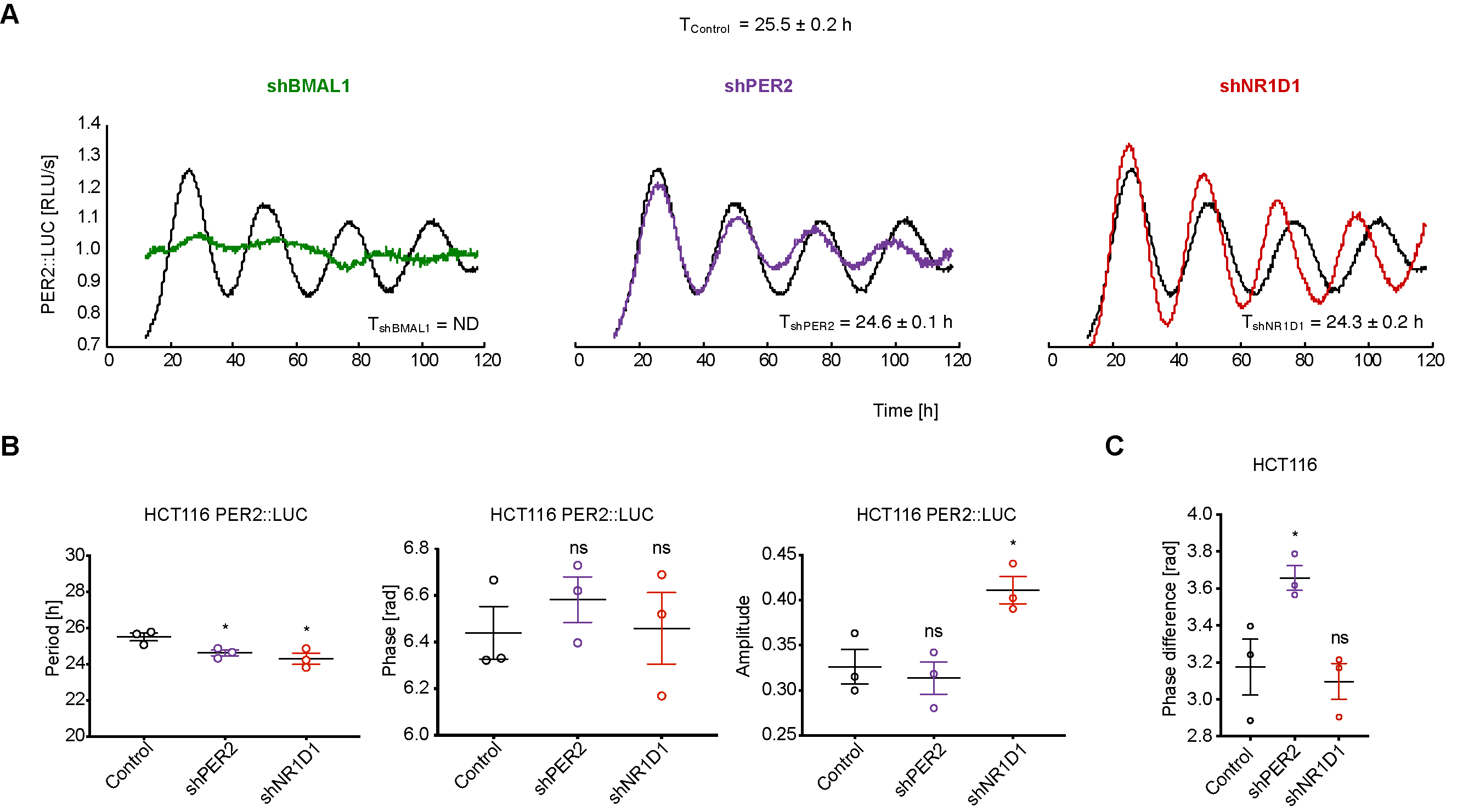
***

**Figure S1:** *PER2* promoter activity show different oscillation patterns in HCT116 knockdown cell lines. (A) Bioluminescence readouts for the promoter activity of *PER2* over the course of 120 hours in HCT116 control (pLKO.1) and knockdown (*shBMAL1*, *shPER2* and *shNR1D1*) cell lines. Periods were calculated with ChronoStar software (T_Control_ = 25.5 ± 0.2 h, T_shBMAL1_ = ND, T_shPER2_ = 24.6 ± 0.1 h, T_shNR1D1_ = 24.3 ± 0.2 h, n=3, mean ± SEM). (B) Period, phase and amplitude analysis of circadian bioluminescence data of HCT116 knockdown cells over the course of 120 hours using Chronostar. (C) Absolute phase-shit between *BMAL1* and *PER2* oscillations for the shPER2 and shNR1D1 knockdown cells compared to the control cell line. ND not defined, ns p > 0.05, *p < 0.05, **p < 0.01, ***p < 0.001; two-tailed unpaired t-test.


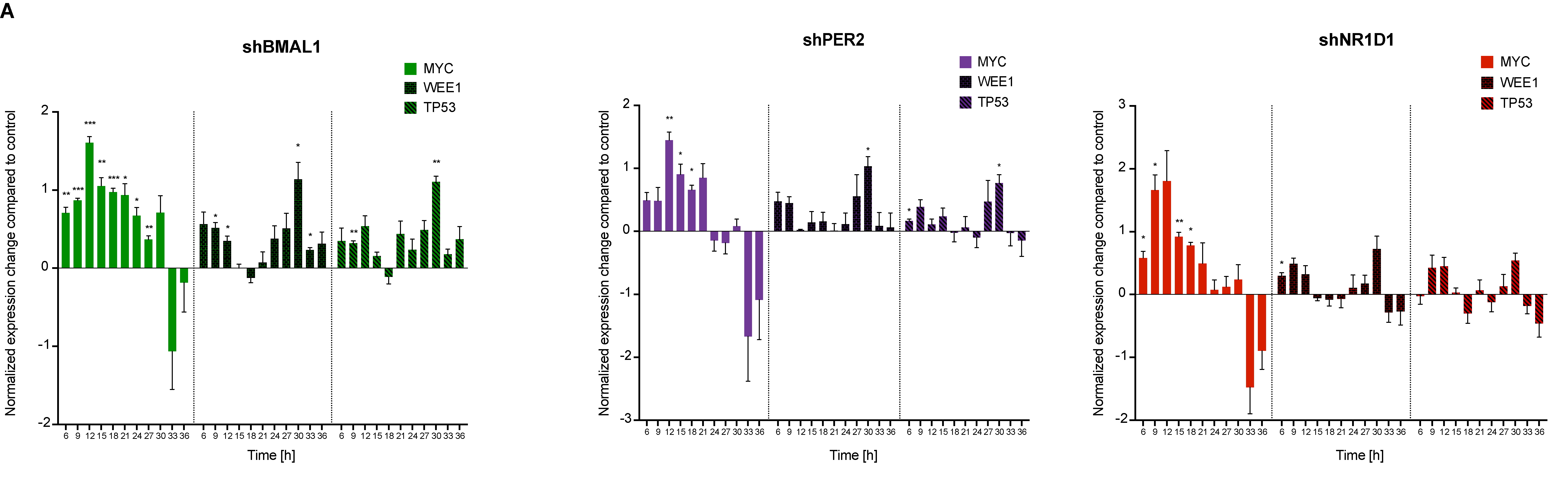


**Figure S2:** 30-hour time-course gene expression analysis for MYC, WEE1 and TP53 in different HCT116 KD cells. (A) Changes in *MYC*, *WEE1* and *TP53* expression over time in HCT116 KD cells compared to control**.** At each time-point, expression of *MYC*, *WEE1* and *TP53* in each KD cell line is compared to the control cell line for a total duration of 30 hours. *p < 0.05, **p < 0.01, ***p<0.001; two-tailed unpaired t-test, adjusted p-values corrected for multiple comparisons using Holm-Sidak method.


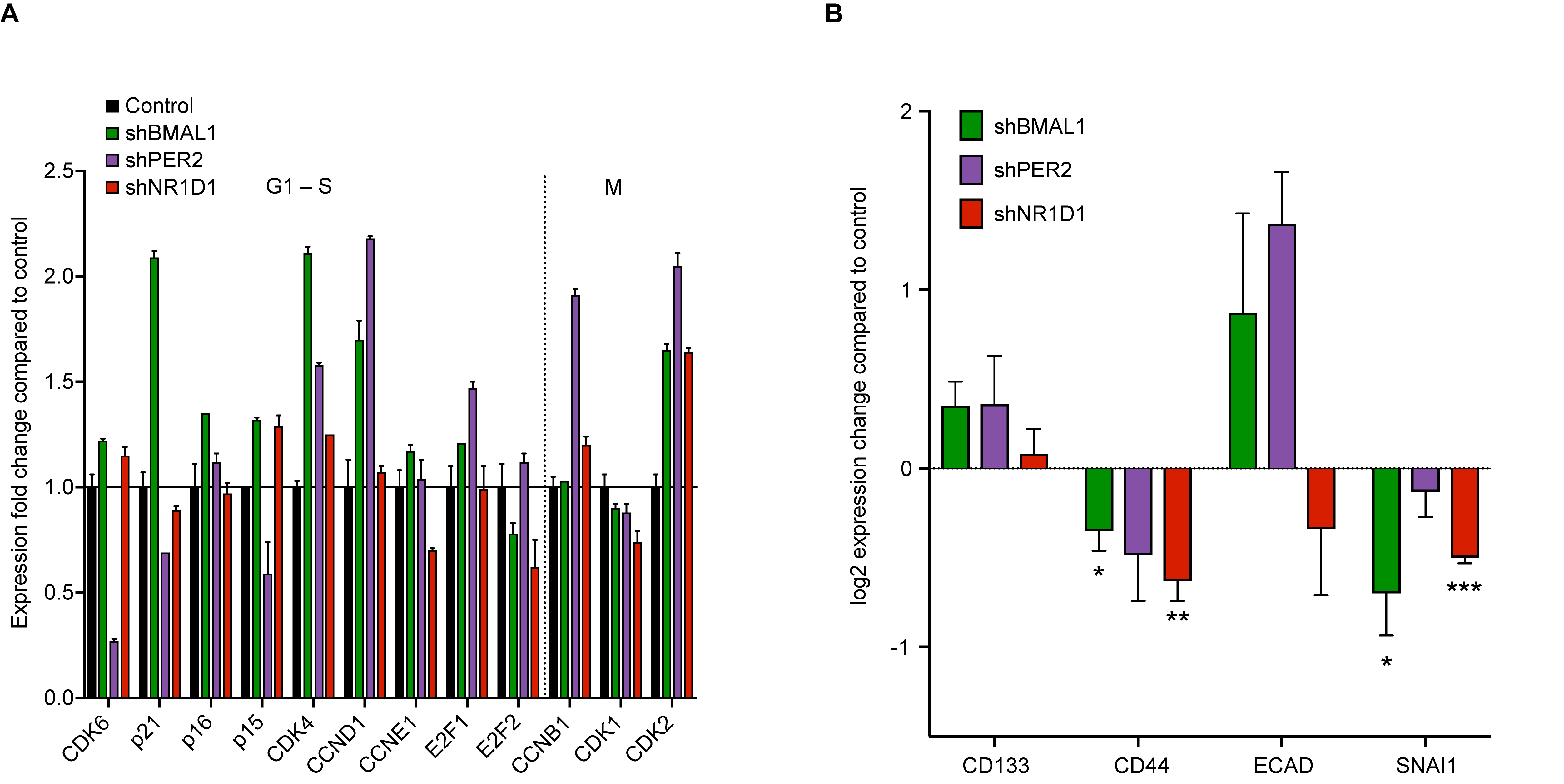


**Figure S3:** Gene expression analysis of: (A) cell cycle related genes and (B) genes involved in EMT. *p < 0.05, ** p< 0.01, ***p < 0.001; two-tailed unpaired t-test.

*
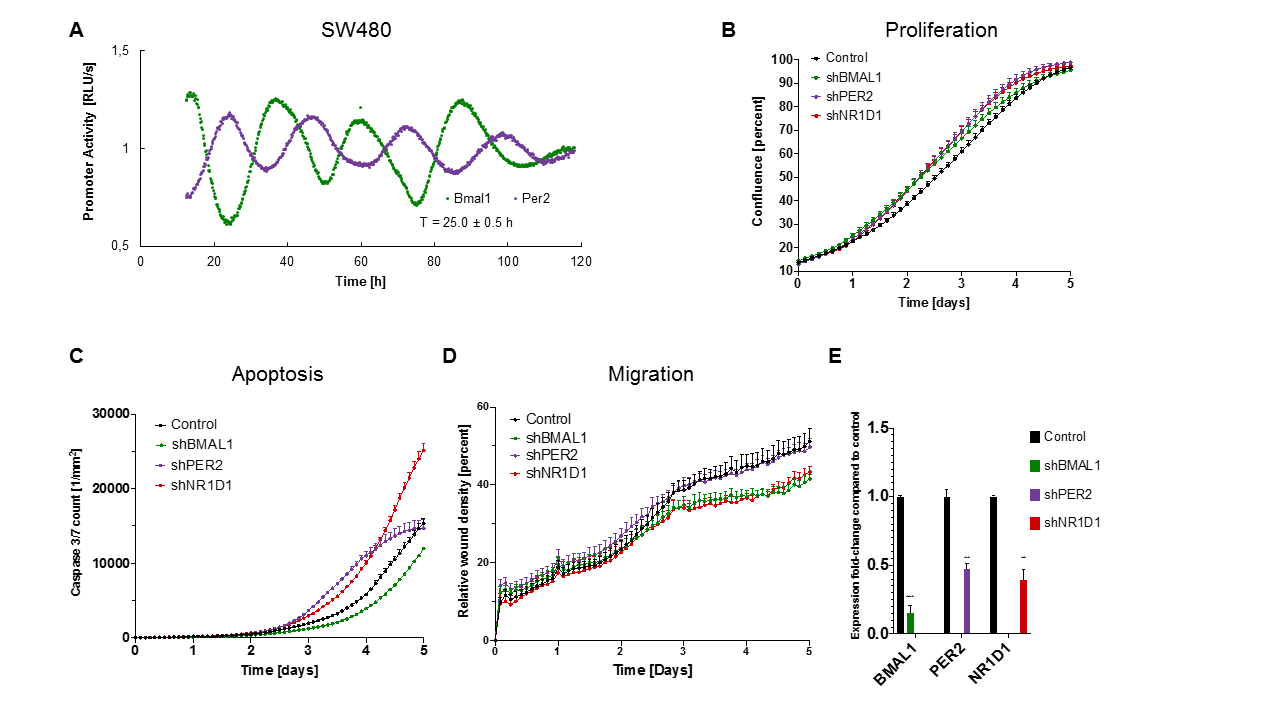
*

**Figure S4:** Core-clock gene knockdown alters cellular properties of SW480 colon cancer cell line. (A) Gene expression analysis of: (A) Bioluminescence readouts for the promoter activity of *BMAL1* and *PER2* over the course of 120 hours in SW480 WT cells. Period was calculated with ChronoStar software (T = 25.0 ± 0.5 h, n=3, mean ± SEM). (B) Proliferation analyses of SW480 cell lines after shRNA knock-down of core-clock genes (*BMAL1*, *PER2* and *NR1D1*) over 5 days. (C) Apoptosis analysis of SW480 cell lines after shRNA KD of *BMAL1*, *PER2* and *NR1D1* (n >8, mean ± SEM). Measurements obtained by counting caspase3/7 green objects per mm^2^ every 2 hours in the course of 5 days using the IncuCyte S3 device. (D) Migration properties of control and shRNA KD SW480 cell lines (*shBMAL1*, *shPER2* and *shNR1D1*). Measurements were obtained using a scratch wound assay (IncuCyte). Quantification was performed by measuring the relative wound density over the course of 5 days. (n >5, mean ± SEM). (E) Gene expression analysis of core-clock genes *PER2*, *NR1D1* and *BMAL1* in SW480 control and knockdown cell lines. *p < 0.05, **p < 0.01, ***p < 0.001; two-tailed unpaired t-test.
